# Effects of Antarctic Krill Meal in Diet on Reproductive Performance and Embryo Quality of *Eriocheir sinensis*

**DOI:** 10.1155/2024/9936529

**Published:** 2024-01-31

**Authors:** Xiaorong Huang, Ru Tan, Zhi Geng, Tao Zhang, Guangpeng Feng, Gang Yang, Feng Zhao, Ping Zhuang

**Affiliations:** ^1^Ministry of Agriculture and Rural Affairs, Key Laboratory of East China Sea and Oceanic Fishery Resources Exploitation and Utilization, East China Sea Fisheries Research Institute, Chinese Academy of Fishery Sciences, Shanghai, China; ^2^Shanghai Engineering Research Center of Fisheries Stock Enhancement and Habitat Restoration of the Yangtze Estuary, Shanghai, China

## Abstract

A 60-day feeding trial was conducted to evaluate the impact of dietary Antarctic krill meal on the reproductive performance and embryo quality of the Chinese mitten crab, *Eriocheir sinensis*. Three diets were formulated, incorporating varying levels of Antarctic krill meal at 0% (Diet K0), 10% (Diet K10), and 20% (Diet K20), with a control group fed razor clam *Sinonovacula constricta*. Each diet was randomly assigned to three replicate tanks, each stocked with 5 males and 10 females. Male and female weights were 145.38 ± 8.01 and 102.57 ± 9.73 g, respectively. The results revealed no significant differences in weight gain rate, specific growth rate, and survival rate. However, the hepatopancreatic weight and hepatopancreas index of female crabs in each group decreased, while gonadal weight and gonadosomatic index increased significantly after 60 days, with Diet K20 showing the highest values. Egg production and fecundity of female crabs reached their peak in Diet K20, with no significant differences in reproductive indices among all groups. The phospholipid content in Diet K20 was significantly higher than in the other groups (*P* < 0.05). Cholesterol contents in Diet K0 and the control group were significantly higher than in Diet K10 and K20 (*P* < 0.05). No significant differences were observed in egg diameter, egg weight, moisture, crude protein, and crude fat between the groups. The content of C20 : 2 and C20 : 4n6 was highest in Diet K0, with a significant difference compared to Diet K10 (*P* < 0.05). However, no significant differences were found in the total content of saturated fatty acids, monounsaturated fatty acids, and polyunsaturated fatty acids among all groups. Based on the research findings, it is recommended that the optimal level of Antarctic krill meal in diets is 20%.

## 1. Introduction

The Chinese mitten crab, *Eriocheir sinensis*, holds significant importance in Chinese aquaculture, contributing to an annual production of 782,200 tons valued at US$ 4.5 billion in 2022 [1]. This species exhibits migratory behavior in its life cycle, with adult crabs predominantly residing in fresh and brackish waters but migrating to estuaries for reproduction [2]. The Yangtze River Estuary is particularly crucial as a breeding ground for Chinese mitten crabs. However, the natural population of this species in China has experienced a sharp decline since the 1980s due to overfishing, water pollution, and the impediment of migration caused by dam and floodgate construction [3]. Studies on the proliferation and release of adult crabs have been proposed to counteract this decline and restore Chinese mitten crab resources in the Yangtze River. Prior research indicates that strengthening the nutrition of released parent crabs before release can enhance their survival rate (SR) and fecundity, thus improving proliferation and release outcomes [4].

It is widely recognized that the nutritional status of crustaceans significantly influences ovarian maturation, reproductive performance, egg quality, and offspring [5–8]. Investigating the nutritional requirements of Chinese mitten crab could lead to enhanced reproductive output and larval quality [3, 9, 10]. Conventional practices in commercial hatcheries involve feeding Chinese mitten crab broodstock a diet comprising fresh foods such as trash fish, razor clam, and sandworm. Razor clam, in particular, is rich in phospholipids (PL), highly unsaturated fatty acids (HUFA), and essential amino acids (EAA), making it a popular choice in Chinese mitten crab hatcheries for promoting gonadal development and improving female reproductive performance [9]. However, natural diets come with high costs, storage challenges, and difficulties in nutrient absorption, which may have negative impacts when supplementing specific nutrients [11, 12].

The Antarctic krill, *Euphausia superba*, plays a pivotal role in the Antarctic ecosystem, boasting an estimated biomass of 100–500 million metric tons [13]. Antarctic krill is abundant in amino acids (aspartate, glutamate, lysine, and leucine) and fatty acids (mainly including C16 : 0, C18 : 1n-9, C20 : 5n3, and C22 : 6n3) [14, 15]. It has been utilized as a protein source in the diets of various aquatic species, including White Prawn *Penaeus vannamei* [16, 17], Swimming Crab *Portunus trituberculatus* [18], Olive flounder (*Paralichthys olivaceus*) [19], Turbot *Scophthalmus maximus* [20], and Large Yellow Croaker *Larimichthys crocea* [21]. However, for crustaceans to be incorporated into fish diets in the future, it is essential that they do not compromise or adversely impact the final product's quality. A reported improvement in the spawning rate of *Litopenaeus vannamei* broodstock with artificial formula feed supplemented with 10%–20% Antarctic krill meal suggests that it can serve as a nutrition enhancer [22]. This paper represents the first study investigating the effects of Antarctic krill meal in the diet on the reproductive performance and embryo quality of the Chinese mitten crab. The results are expected to offer theoretical references and scientific support for enhancing broodstock in the artificial propagation and release of the Chinese mitten crab.

## 2. Materials and Methods

### 2.1. Preparation of Diets

Three diets were developed, incorporating varying levels of Antarctic krill meal at 0% (Diet K0), 10% (Diet K10), and 20% (Diet K20), as outlined in the literature [22]. The razor clam, renowned for its high nutritional value, is commonly utilized in the commercial breeding of the Chinese mitten crab. To assess the nutritional enhancement effects of formulated versus natural diets, the razor clam was chosen as the control group. The formulation and composition of each diet are detailed in Table 1. The ingredients of the experimental diets were thoroughly mixed and extruded into pellets with a diameter of 4 mm, utilizing a pellet extruder from Dajiang Feed Co., Ltd, Changzhou, China. Subsequently, the experimental diets were dried at room temperature.

### 2.2. Broodstock Rearing

Adult male and female Chinese mitten crabs were procured from Qidong, Jiangsu Province, China, in early October, with initial body weights (BWs) of 145.38 ± 8.01 g for males and 102.57 ± 9.73 g for females. The crabs were transported to the laboratory at the East China Sea Fisheries Research Institute, Chinese Academy of Fishery Sciences, Shanghai, China. Prior to the commencement of the experiment, male and female crabs underwent a 1 week acclimation period in separate fiberglass water tanks (diameter 2.0 m). Three experimental groups (K0, K10, and K20 diets) and one control group were established, each comprising three replicate tanks with 10 crabs. The experiment took place in circular tanks with a diameter of 1.5 m and a water depth of 60 cm. About 40% of the tank's bottom area was covered with fine sand (10–20 cm), and polyvinyl chloride tubes (diameter 15 cm) were provided as shelters for the crabs. Throughout the experiment, all rearing tanks adhered to a natural photoperiod of approximately 12 hr of light and 12 hr of darkness. Crabs were fed daily at 6 : 00 PM, with the amount provided at 3%–5% of their live BW. Food residues were removed the following morning. Daily measurements of water temperature in each tank were taken at 8 : 00 AM and 8 : 00 PM. Additionally, ammonia-N, nitrite, dissolved oxygen, and pH levels were monitored every 2 days.

### 2.3. Reproductive Performance

The reproductive experiment involved stocking five males and 10 females in a tank after a 60-day feeding period, with each treatment having three replicate tanks. The water salinity was maintained at 18 ppt based on pre-experimental results [23]. Observations of the mating status of adult crabs were conducted daily in each experimental group. The wet weights of female crabs were initially measured, followed by the dissection and weighing of ovaries and hepatopancreas on both the 1st and 60th days. Gonadosomatic index (GSI) and hepatopancreas index (HSI) were calculated using established methods as follows:

HSI (%) = 100 × hepatopancreas wet weight/body wet weight;

GSI (%) = 100 × gonad wet weight/body wet weight.

After spawning, nine crabs at the fertilized egg stage were collected from each treatment, and their wet weights were determined after surface moisture absorption with filter paper. To estimate the total number of eggs per female (eggs/female), three subsamples of eggs from each spawning crab were weighed, and the total number of eggs in each sample was calculated using an optical microscope (Olympus). Fecundity, defined as the number of eggs per gram of female weight, was calculated by dividing the total egg number by the weight of the female. The reproductive index (100 × egg clutch weight g^−1^ female) was determined by dividing the eggs clutch wet weight by the BW of a female crab. The diameter of 80 randomly selected eggs from each spawning crab was measured using an optical microscope (Olympus).

For egg dry weight (DW) measurements, subsamples of eggs were subjected to a 70°C oven for 48 hr, following the method described by Cavalli et al. [24]. The remaining embryos were stored at −80°C for subsequent biochemical analysis.

### 2.4. Chemical Analysis

The analyses of moisture content, crude protein (Kjeldahl method, utilizing a 6.25 N to protein conversion factor), and ash content were carried out following AOAC procedures. Total lipid was extracted with chloroform: methanol (2 : 1, v/v) as per the method described by Floch et al. [25]. Phospholipid (PL) separation was achieved using petroleum: methanol (1 : 1, v/v), while triglyceride (TG) and cholesterol (CHL) were analyzed using the two-step development system outlined by Skipski et al. [26]: (1) isopropyl ether-acetic acid, 96 : 4 (v/v); and (2) petroleum ether-diethyl ether-acetic acid, 90 : 10: 1 (by vol.). Fatty acid methyl esters (FAME) were prepared through transesterification with 0.4 M KOH-methanol. Analytical verification of FAME was performed using flame ionization detection after sample injection into an Agilent 6890 gas chromatograph equipped with an HP-5.5% Phenyl Methyl Siloam capillary column. Injector and detector temperatures were set at 300°C. The column temperature was initially held at 60°C for 2 min, followed by a rate increase of 20°C/min to 150°C, then to 280°C at a rate of 4°C/min, where it was held until all FAME had been eluted. Helium served as the carrier gas with a flow velocity of 40 ml/min. Elution peaks were identified by comparing retention times with known standards (Nu-Chek-Prep, Elysian, MN, USA). Fatty acid contents were expressed as the percentage of each fatty acid to the total fatty acid content (%).

### 2.5. Statistical Analysis

Data are presented as mean ± standard deviation (SD). Statistical analyses were conducted using analysis of variance, and the Tukey test was employed in this study. A significance level of *P* < 0.05 was considered statistically significant for any two treatments. All statistical analyses were performed using the SPSS statistics package software (version 22.0).

## 3. Results

### 3.1. Chemical Composition of Experiment Diets

The proximate nutrient composition, total protein (TP), HUFA, and fatty acid contents of each formulated diet and the razor clam are presented in Table 2. As anticipated, the razor clam exhibited a significantly higher moisture content (87.10%) compared to each formulated diet. Moreover, higher levels of crude protein, crude lipid, crude fiber, ash, and TP were measured in each formulated diet in comparison to the razor clam. In terms of fatty acid contents, elevated levels of C18 : 2n6, C20 : 4n3, C20 : 5n3, C22 : 6n3, and HUFA were observed in all formulated diets as compared to the razor clam.

### 3.2. Growth, HSI, and GSI of the Female Crabs

After a 60-day feeding period with four diets, the final weight of the K0 group showed no significant difference from the initial value, while the final weights of the other three groups were significantly higher than their respective initial weights (*P* < 0.05). There were no significant differences observed in weight gain rate (WGR), specific growth rate (SGR), and SR among the groups. The hepatopancreatic weight (HW) and hepatosomatic index (HSI) of female crabs in each diet group were significantly lower than the initial values (*P* < 0.05). The K0 group exhibited the lowest values, although no significant differences were observed among the groups. On the other hand, gonadal weight (GW) and GSI in each experimental group were significantly higher than the initial values (*P* < 0.05). The K20 group had the highest values, and there were significant differences among the experimental groups (refer to Table 3).

### 3.3. Reproductive Performance of the Female Crabs

**Figure 1:**
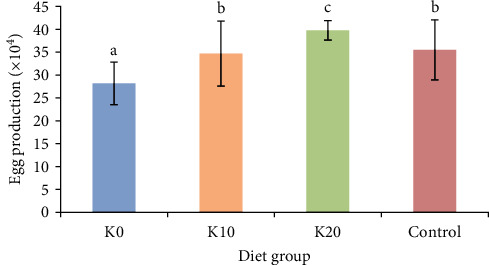
Effects of feeding four diets on egg production of female crabs. *Note*. Diet K0, Diet K10, and Diet K20 represent diets in which Antarctic krill meal is 0%, 10%, and 20%, respectively. Control represent diet which fed with the razor clam. Egg production was represented using total number of eggs per female, different lowercase letters represent significant difference in egg production of female crabs (*P* < 0.05, *n* = 9).

Female crabs in the K0 group exhibited the lowest egg production, averaging (28.17 ± 4.65) × 10^4^ eggs per crab (Figure 1). In contrast, female crabs in the K20 group demonstrated the highest egg production, averaging (39.78 ± 2.16) × 10^4^ eggs per crab, significantly surpassing the other salinity groups (*P* < 0.05). The egg production of female crabs in the K10 group and the control group was relatively similar, with no significant difference between them.

**Figure 2:**
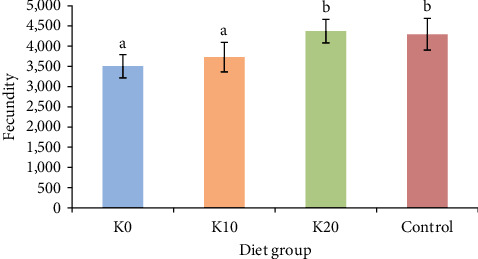
Effects of feeding four diets on the fecundity of female crabs. *Note*. Diet K0, Diet K10, and Diet K20 represent diets in which Antarctic krill meal is 0%, 10%, and 20%, respectively. Control represents a diet which fed with the razor clam. Fecundity is defined as the number of eggs per gram of female weight, and different lowercase letters represent significant differences in the fecundity of female crabs (*P* < 0.05, *n* = 9).

The highest fecundity among female crabs was observed in those fed the K20 diet, slightly exceeding the control group, although no significant difference was detected between them (Figure 2). The K0 group exhibited the lowest fecundity, averaging 3,503.37 ± 287.61 eggs per gram, but no significant difference was observed compared to the K10 group.

**Figure 3:**
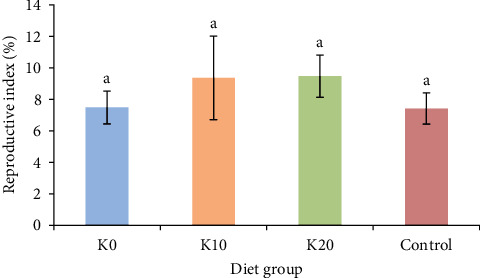
Effects of feeding four diets on the reproductive index of female crabs. *Note*. Diet K0, Diet K10, and Diet K20 represent diets in which Antarctic krill meal is 0%, 10%, and 20%, respectively. Control represents a diet which fed with the razor clam. The reproductive index (100 × egg clutch weight/g female) was calculated by dividing the egg clutch wet weight by the BW of a female crab.

The impact of different feeding groups on the reproductive index of female crabs is illustrated in Figure 3. Reproductive indexes of female crabs fed K10 and K20 were greater than those of females fed K0 and razor clam. However, there were no significant differences observed among all experimental groups.

### 3.4. Egg Diameter and Weight of Embryos

The egg diameter of female crabs ranged from 357 to 362 *μ*m in all treatments (Table 4), and no significant difference in egg diameter was observed among the treatments. The wet and DWs of eggs were the highest in the K20 group, averaging 47.92 ± 8.01 and 11.68 ± 1.08 *μ*g, respectively, but there were no significant differences among the experimental groups.

### 3.5. Biochemical Compositions of Embryos

Changes in biochemical components in embryos of females fed different diets are presented in Table 5. No significant differences were observed in moisture, crude protein, TP, and crude fat among the four groups. The phospholipid content in Diet K20 was significantly higher than in the other groups (*P* < 0.05).The highest TG content was detected in the K10 group, with an average of 1,449.73 ± 101.38 *μ*g/g, significantly higher than the other groups (*P* < 0.05). The CHL content in the K20 and control groups was relatively similar, with no significant difference between them, but both were significantly higher than those of the K0 and K10 groups (*P* < 0.05).

A total of 18 fatty acids were detected in the embryos, comprising five types of saturated fatty acids (SFA), five types of monounsaturated fatty acids (MUFA), and eight types of polyunsaturated fatty acids (PUFA), as shown in Table 6. The total amount of SFA in the control group was the highest, with an average content of 11.03 ± 3.95 mg/g, but no significant differences were observed among the groups. The content of C16 : 1 and C18 : 1n9c was highest in the control group, with no significant differences among the groups, and no significant differences were observed among the groups in the total amount of MUFA. C20 : 2 and C20 : 4n6 content were both highest in the K0 group, with a significant difference compared to the K10 group (*P* < 0.05), but there was no significant difference between K20 and the control group. The total amount of PUFA was the highest in the control group, averaging 20.86 ± 6.88 mg/g, but no significant difference was observed among all groups, and no significant differences in HUFA were observed among the groups.

## 4. Discussion

Razor clam is considered one of the best natural foods for Chinese mitten crab due to its high levels of HUFA, PL, and EAA [27]. Previous studies have demonstrated that feeding Chinese mitten crab broodstock with razor clam improves reproductive performance [9]. Therefore, it has been widely used in commercial hatcheries of Chinese mitten crab in China [10]. Previous studies have illustrated that Antarctic krill meal supplementation could enhance growth performance and promote feed utilization in crustaceans [27, 28]. In contrast, Nunes et al. [29] and Guo et al. [18] demonstrated that dietary low Antarctic krill meal supplementation had no positive influence on the growth performance of *L. vannamei* and *P. trituberculatus*, respectively. In the present study, there were no significant effects on the growth performance of Chinese mitten crabs among different diet groups. The hepatosomatic index (HSI) and GSI are important reference indicators for evaluating the maturity of Chinese mitten crab. In nature, Chinese mitten crab accumulates energy in the hepatopancreas and develops reproductive organs, leading to an increase in gonads [8]. The HW and HSI of Chinese mitten crab declined significantly; however, there was a significant increase in GW and GSI after feeding for 60 days. The results also confirmed that during the development of the gonads, energy substances in the hepatopancreas were transferred to the gonads, leading to a decrease in HSI and an increase in GSI. These results were in line with the observations by Zhang et al. [22]. The GSI was the highest in the K20 group, followed by the K10 group. K10 and K20 diets contained higher levels of crude protein, crude fat, and total phosphorus (Table 2), providing more abundant nutrients for the Chinese mitten crab broodstock during the feeding stage, resulting in higher GW and GSI. These results also showed that 10%–20% Antarctic krill meal in the diet could promote the gonadal development of female crabs.

The reproductive performance of crustaceans mainly includes egg production, reproductive index, and fecundity, which are crucial indicators for evaluating broodstock quality [30]. Broodstock reproductive performance is influenced by various factors, including diet, gonadal development, specifications, water temperature, and others [10]. The content and composition of PL and unsaturated fatty acids in diets can impact the reproductive performance of female Chinese mitten crabs [12]. The egg production and fecundity of Chinese mitten crabs improve with increasing dietary PL [3]. In the present study, the egg production and fecundity of female crabs in the K20 group were higher than those of other groups. This may be attributed to the highest total phosphorus and important fatty acid content in the K20 group, providing sufficient nutrition for the gonadal development of female crabs and resulting in the highest egg production and fecundity. These results align with earlier studies on penaeid shrimp [31, 32] and Chinese mitten crab [12]. However, the reproductive index of female crabs did not show significant differences among the treatments.

There was no significant difference in egg size and single egg weight among embryos produced by different crustacean broodstock, such as *L. vannamei, M. rosenbergii*, and *P. trituberculatus* [33–35]. Similarly, there was no significant difference in egg diameter and weight between cultured and wild Chinese mitten crab broodstock with similar sizes, indicating that egg diameter and weight are relatively stable and not easily influenced by growth conditions [23]. In the present study, there were no significant differences in the diameter and weight of eggs among the four groups after nutrient enrichment with different diets. The research results were essentially consistent with the aforementioned reports.

Proteins and lipids are crucial energy sources during the embryonic development of crustaceans, and the biochemical components of embryos can reflect the storage and utilization of energy substances [36, 37]. The content of nutrients in embryos is mainly influenced by the accumulation level of maternal nutrients before spawning and the utilization of nutrients during embryonic development [38]. In this study, although there were certain differences in the content of crude protein and crude fat among the four diets, there were no significant differences in crude protein, crude fat, and TP among the embryos in each group. This may be partly explained by selective absorption and preferential accumulation of nutrients during nutritional enhancement and embryonic development [9, 12]. PL are considered essential nutrients in many crustacean diets and play a vital role in the transfer of lipids from the hepatopancreas to the ovary during ovarian development [5]. The phospholipid content in embryos was highest in the K20 group, and simultaneously, the gonadal index was also the highest after feeding the K20 diet. This indicates the transfer of PL from the gonads to the embryos, resulting in a higher phospholipid content. These results confirm that a 20% Antarctic krill meal in the diet can significantly increase the phospholipid content in embryos.

The maturation of gonads and reproduction in crustaceans require a substantial amount of PUFA, which crustaceans cannot synthesize on their own and must obtain from their diet [39, 40]. The appropriate content and proportion of HUFA in crustacean ovaries and embryos are crucial for supporting embryo development and can significantly improve the SR and metamorphosis rate of newly hatched larvae [12]. Antarctic krill meal has a high content of EPA (eicosapentaenoic acid) and DHA (docosahexaenoic acid), with contents as high as 21.42% and 19.22%, respectively. The n-3 series and n-6 series fatty acids account for 45.41% and 2.24%, respectively [41]. Dietary supplementation with Antarctic krill meal increased the content of EPA and n-3 PUFA while decreasing n-6 PUFA content in tongue sole embryos [42]. The composition of egg fatty acids is positively correlated with the composition of fatty acids in the diet. The PUFA content in the group fed with silkworm was the highest, followed by the group fed with 10% Antarctic krill meal and the 20% group [22]. In the present study, although the content of C20 : 4n3, C20 : 5n3, and C22 : 6n3 was highest in diet K20, there was no significant difference in the content of C20 : 5n3, C22 : 6n3, SFA, MUFA, PUFA, and HUFA in embryos after nutrient enrichment with different diets, and these results were generally consistent with those of *L. vannamei* [22]. There was no significant difference between ∑PUFA and ∑HUFA in Chinese mitten crab embryos after feeding a formulated diet and razor clam separately [8]. These results indicate that the addition of Antarctic krill meal in the diet and feeding with razor clam cannot significantly increase the content of fatty acids in the embryos of Chinese mitten crab.

## 5. Conclusion

The findings indicate that incorporating 10%–20% Antarctic krill meal into the diet significantly promotes the gonadal development of female Chinese mitten crab. Moreover, a diet containing 20% Antarctic krill meal enhances egg production and fecundity in female Chinese mitten crabs, along with an increase in the contents of phospholipid and CHL in embryos. However, the addition of Antarctic krill meal to the diet does not lead to a significant increase in the content of fatty acids in embryos. This study provides valuable scientific support for the artificial propagation and release of Chinese mitten crab.

## Figures and Tables

**Table 1:** Formulation and composition of experimental diets (%).

Ingredients	Diet K0	Diet K10	Diet K20
Antarctic krill meal	—	10.0	20.0
Fish meal	40.0	40.0	40.0
Soybean meal	18.0	18.0	18.0
Casein	8.2	5.5	3.8
Wheat gluten meal	23.25	15.65	7.15
Mineral premix	1.0	1.0	1.0
Vitamin premix	0.5	0.5	0.5
Fish oil	6.0	6.0	6.0
Choline chloride	0.5	0.5	0.5
Ca(H_2_PO_4_)_2_	0.5	0.5	0.5
Inositol	0.5	0.5	0.5
Cholesterol	0.4	0.4	0.4
Vitamin C	0.65	0.65	0.65
Astaxanthin	0.5	0.8	1.0
Total	100.00	100.00	100.00

*Note*: Diet K0, Diet K10, and Diet K20 represent diets in which Antarctic krill meal is 0%, 10%, and 20%, respectively. Fish meal, soybean meal, casein, wheat gluten meal, and fish oil: from Shanghai, Yongnong Feed, Minhang, Shanghai, China; Inositol: from Shanghai Chemical Reagent, Huangpu, Shanghai, China; Monocalcium phosphate: from Sinopharm Chemical Reagent Co., Ltd., China; Choline chloride, vitamin C, and astaxanthin: purchased from Aladdin; Mineral premix: 1 kg of diet contained Ca(H_2_PO4)_2_, 10 g; MgSO_4_ · 7H_2_O, 2.4 g; KCl, 4.5 g; NaCl, 2.1 g; FeSO_4_ · H_2_O, 155 mg; CuSO_4_ · 5H_2_O 40 mg; ZnSO_4_ · H_2_O 80 mg; MnSO_4_ · H_2_O 30 mg; KI, 11.7 mg; CoCl_2_ · 6H_2_O 4.8 mg; Na_2_SeO_3_, 2.4 mg. Vitamin premix (mg/kg diet): vitamin A, 32; vitamin E, 200; vitamin D3, 0.05; vitamin B1, 30; vitamin B2, 30; vitamin B6, 20; vitamin B12, 0.1; nicotinic acid, 200; folic acid, 15; biotin, 3; calcium pantothenate, 100.

**Table 2:** Nutrient composition and fatty acid content of each diet.

Items	Diet group			
	Diet K0	Diet K10	Diet K20	Control
Crude protein (%)	37.70	42.89	42.20	7.05
Crude lipid (%)	5.40	6.20	6.80	0.30
Crude fiber (%)	5.40	5.20	5.20	0.90
Ash (%)	11.1	11.30	11.50	1.40
Total phosphorus (*µ*g/g)	1.48 × 10^4^	1.94 × 10^4^	2.01 × 10^4^	1.04 × 10^3^
Moisture (%)	10.3	6.74	6.84	87.10
C18 : 2n6 (%)	5.62	4.37	3.45	0.49
C18 : 3n3 (%)	1.65	1.36	1.82	1.48
C20 : 3n3 (%)	0.45	0.64	0.78	0.36
C20 : 3n6 (%)	0.06	0.13	0.24	0.04
C20 : 4n3 (ARA) (%)	2.28	5.04	6.17	1.54
C20 : 5n3 (EPA) (%)	6.82	8.56	9.43	4.37
C22 : 6n3 (DHA) (%)	9.21	12.83	14.16	3.86
Total HUFA (%)	18.31	26.43	29.76	9.77

*Note*: Diet K0, Diet K10, and Diet K20 represent diets in which Antarctic krill meal is 0%, 10%, and 20%, respectively. Control represents diet, which is fed with the razor clam. Total HUFA = ARA + EPA + DHA.

**Table 3:** Growth, HSI, and GSI of female crabs in different diet groups.

Parameters	The initial value	Diet group			
		K0	K10	K20	Control
BW (g)	102.57 ± 9.73^a^	116.04 ± 7.53^ab^	118.02 ± 5.48^b^	119.18 ± 8.53^b^	118.43 ± 6.18^b^
WGR (%)	—	13.13 ± 3.54	15.06 ± 4.40	16.19 ± 4.15	15.46 ± 4.62
SGR	—	0.21 ± 0.04	0.24 ± 0.05	0.25 ± 0.04	0.24 ± 0.05
SR (%)	—	93.14 ± 1.58	94.37 ± 2.16	93.85 ± 1.43	92.26 ± 0.98
Hepatopancreatic weight (g)	7.79 ± 1.03^a^	3.81 ± 0.39^b^	4.50 ± 0.91^b^	6.20 ± 0.41^b^	5.24 ± 1.42^b^
HSI (%)	8.71 ± 0.84^a^	5.15 ± 1.02^b^	5.49 ± 1.02^b^	5.75 ± 2.34^b^	6.29 ± 1.32^b^
Gonadal weight (g)	5.51 ± 1.87^a^	9.18 ± 1.17^b^	10.35 ± 0.89^b^	12.04 ± 0.47^c^	9.96 ± 0.94^b^
GSI (%)	6.18 ± 2.19^a^	12.32 ± 1.92^b^	12.72 ± 1.66^b^	14.84 ± 1.38^c^	12.16 ± 1.52^b^

*Note*: Data in the table are represented as means ± SD (*n* = 9). BW, body weight; WGR, weight gain rate; SGR, specific growth rate; SR, survival rate. HSI, hepatopancreas index; HSI (%) = 100 × hepatopancreas wet weight/body wet weight; GSI, gonadosomatic index; GSI (%) = 100 × gonad wet weight/body wet weight; Tukey test of statistical analysis is used to compare the means, values in each row with different superscripts are significantly different (*P* < 0.05).

**Table 4:** Comparison of egg diameter and egg weight of female crabs.

Parameters	Diet group			
	K0	K10	K20	Control
Egg diameter (*μ*m)	357.34 ± 9.30	360.81 ± 11.91	362.75 ± 8.35	362.13 ± 9.22
Egg wet weight (*μ*g)	42.83 ± 6.17	46.39 ± 5.10	47.92 ± 8.01	43.44 ± 2.11
Egg dry weight (*μ*g)	11.33 ± 1.28	11.65 ± 1.11	11.68 ± 1.08	11.59 ± 1.46

*Note*: Data in the table are represented as means ± SD (*n* = 9). Egg diameter was measured using an optical microscope, egg wet weight was measured by weighing, and egg dry weight was measured by oven drying. Tukey test of statistical analysis is used to compare the means; values in each row with different superscripts are significantly different (*P* < 0.05).

**Table 5:** Biochemical composition in embryos of females fed different diets.

Parameters		Diet group		
	K0	K10	K20	Control
Moisture (%)	65.17 ± 2.10	67.23 ± 1.31	67.18 ± 2.37	67.18 ± 2.24
Crude protein (%)	17.23 ± 0.38	18.07 ± 0.25	18.12 ± 0.50	18.22 ± 0.81
Crude fat (%)	9.03 ± 0.91	9.79 ± 0.28	9.72 ± 0.18	9.80 ± 0.09
Triglyceride (*μ*g/g)	1,189.77 ± 80.55^a^	1,449.73 ± 101.38^b^	1,116.73 ± 61.37^a^	1,155.37 ± 91.08^a^
Cholesterol (*μ*g/g)	1,647.37 ± 67.19^a^	1,270.90 ± 69.29^b^	1,097.62 ± 47.32^b^	1,647.24 ± 109.73^a^
Phospholipid (pg/g)	1,189.89 ± 60.13^a^	1,117.66 ± 85.19^b^	1,451.41 ± 80.45^c^	1,157.13 ± 91.08^b^
Total protein (*μ*g/g)	81,752.24 ± 2,841.52	79,354.99 ± 3,036.49	76,425.03 ± 3,094.70	77,961.73 ± 5,396.86

*Note*: Data in the table are represented as means ± SD (*n* = 9). The Tukey test of statistical analysis is used to compare the means. Values in each row with different superscripts are significantly different (*P* < 0.05).

**Table 6:** Fatty acid composition of embryos in different diet groups (mg/g wet weight).

Parameters	Diet group			
	K0	K10	K20	Control
C14 : 0	0.47 ± 0.18	0.34 ± 0.01	0.50 ± 0.09	0.60 ± 0.35
C15 : 0	0.18 ± 0.06^ab^	0.13 ± 0.01^b^	0.17 ± 0.01^ab^	0.28 ± 0.09^a^
C16 : 0	8.04 ± 2.89	6.33 ± 1.51	7.49 ± 1.03	9.77 ± 4.21
C17 : 0	0.29 ± 0.11	0.18 ± 0.06	0.22 ± 0.06	0.31 ± 0.08
C18 : 0	2.05 ± 0.73	1.67 ± 0.13	1.84 ± 0.12	2.37 ± 1.19
∑SFA	11.03 ± 3.95	8.65 ± 1.56	10.22 ± 1.04	13.34 ± 5.78
C16 : 1	7.07 ± 2.77	6.82 ± 3.71	5.24 ± 1.09	7.32 ± 3.20
C20 : 1	0.23 ± 0.08	0.15 ± 0.04	0.23 ± 0.09	0.26 ± 0.12
C24 : 1	0.08 ± 0.01	0.06 ± 0.02	0.06 ± 0.01	0.10 ± 0.04
C18 : 1n9c	15.11 ± 4.46	13.96 ± 4.94	14.69 ± 1.65	17.64 ± 6.75
C22 : 1n9	0.07 ± 0.01	0.06 ± 0.01	0.06 ± 0.01	0.08 ± 0.01
∑MUFA	22.56 ± 7.30	21.04 ± 8.70	20.25 ± 2.06	25.39 ± 9.51
C18 : 2n6c	6.73 ± 2.31	7.02 ± 2.36	5.90 ± 0.74	7.02 ± 3.24
C18 : 3n3	1.26 ± 0.68	1.11 ± 0.10	0.82 ± 0.21	1.40 ± 0.61
C20 : 2	0.63 ± 0.17^a^	0.31 ± 0.06^b^	0.35 ± 0.12^ab^	0.45 ± 0.08^ab^
C20 : 3n3	0.16 ± 0.04	0.08 ± 0.03	0.11 ± 0.04	0.17 ± 0.09
C20 : 3n6	0.09 ± 0.02	0.06 ± 0.01	0.06 ± 0.01	0.09 ± 0.03
C20 : 4n6 (ARA)	2.38 ± 0.69^a^	1.24 ± 0.38^b^	1.55 ± 0.31^ab^	2.31 ± 0.20^ab^
C20 : 5n3 (EPA)	4.75 ± 1.73	3.01 ± 0.47	4.86 ± 0.74	4.85 ± 1.66
C22 : 6n3 (DHA)	3.71 ± 1.73	1.94 ± 1.09	4.18 ± 2.96	4.59 ± 2.15
∑PUFA	19.71 ± 6.95	14.75 ± 1.79	17.82 ± 1.37	20.86 ± 6.88
∑HUFA	10.85 ± 3.94	6.18 ± 0.92	10.59 ± 1.98	11.74 ± 3.98

*Note*: Data in the table are represented as means ± SD (*n* = 3). Tukey test of statistical analysis is used to compare the means; values in each row with different superscripts are significantly different (*P* < 0.05). ∑SFA is the total content of saturated fatty acids; ∑MUFA is the total content of monounsaturated fatty acids, ∑PUFA is the total content of polyunsaturated fatty acids, ∑HUFA = ARA + EPA + DHA.

## Data Availability

Data will be made available on request.
